# Effect of Relaxation‐Based Virtual Reality on Psychological and Physiological Stress of Substance Abusers Under Detoxification: A Randomized Controlled Trial

**DOI:** 10.1002/brb3.70084

**Published:** 2024-10-14

**Authors:** Shahab Lotfinia, Aram Yaseri, Pardis Jamshidmofid, Fatemeh Nazari, Homa Shahkaram, Jafar Sarani Yaztappeh, Amir Sam Kianimoghadam

**Affiliations:** ^1^ Department of Clinical Psychology Taleghani Hospital Research Development Unit School of Medicine Shahid Beheshti University of Medical Science Tehran Iran; ^2^ School of Medicine Shahid Beheshti University of Medical Science Tehran Iran

**Keywords:** relaxation, stress, substance‐related disorders, virtual reality

## Abstract

**Introduction:**

Substance abuse is a widespread problem, with high rates of treatment dropout. Stress plays a crucial role in this problem, so innovative interventions with stressed patients can assist them in completing treatment.

**Methods:**

This study is a randomized controlled trial with 60 participants who have substance abuse disorder undergoing detoxification at a residency facility in Tehran, Iran. Participants were randomly assigned to one of three groups: watching a 360° video of nature, a 360° video of a city environment, or no virtual experience. The intervention was performed only for one session. Psychological stress was measured using the Positive and Negative Affect Schedule and Perceived Stress Scale before and after the intervention. Physiological stress was assessed through respiratory rate, skin conductance, and heart rate recordings during the virtual reality (VR) experience. Data analysis was performed using R software (version 4.2).

**Results:**

Paired *t*‐test results indicated significant psychological differences before and after virtual nature experiences, but not in the control and city groups. The repeated measure ANOVA showed a significant reduction in skin conductance (*p < *0.01) and respiratory rate (*p < *0.01) scores in the nature group.

**Conclusion:**

The findings suggest that VR relaxation could be a potentially beneficial intervention for reducing stress in patients during detoxification.

## Graphical abstract

This study found that using virtual reality to watch natural videos can reduce stress, negative affect, skin conductance, and respiratory rate and increase positive affect in patients with opiate dependency during detoxification.

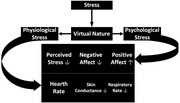

## 1 Introduction

Substance abuse is a major global concern that involves excessive use and addiction to drugs or other substances (Chie et al. 2016), often leading to high dropout rates during treatment (Şimşek, Dinç, and Ögel 2019), particularly during detoxification (Gossling et al. 2001). Detoxification is an essential part of recovery methods (Bergenstrom and Abdul‐Quader 2010), and the primary objective of that is to manage withdrawal symptoms safely and enhance the effectiveness of treatment. Withdrawal symptoms typically subside within 5–14 days, depending on the drug's half‐life (Di Patrizio et al. 2022).

Patients during residential treatment experience severe distress in the first few days after discontinuation for withdrawal symptoms, such as difficulty in inhibiting inappropriate behaviors, delayed reinforcement, and stressed due to the lack of impulse control (Hyman, Paliwal, and Sinha 2007; Sinha 2008). The brain significantly impacts how we respond to stress. A complex neural circuit involving the hippocampus, amygdala, and areas of the prefrontal cortex determines what is deemed threatening and stressful for an individual. These regions work together to regulate physiological and behavioral processes associated with stress. It is important to note that stress processes result from the communication between the brain and the autonomic, cardiovascular, and immune systems, influencing our thoughts, experiences, and actions (McEwen and Gianaros 2010). Patients, who are unable to handle emotional distress, are at a higher risk of leaving the program. The major theories of addiction suggest that stress plays a critical role in addiction processes. Psychological models propose that drug use is a coping strategy to deal with stress, whereas neurobiological models explain how neuroadaptations in reward, learning, and stress pathways may enhance craving, loss of control, and compulsion, leading to addiction (Baker et al. 2004; Hyman and Malenka 2001; Sinha 2008). Furthermore, stress‐induced vulnerability is even more significant than the vulnerability induced by drug cues when it comes to relapse and dropout. As a result, substance users may require additional help to complete the discontinuation process successfully and remain in treatment (Jarvis et al. 2018; Mattick et al. 2014).

Studies have demonstrated that being in natural surroundings can positively impact both physical and mental well‐being. Spending time in nature can promote relaxation, reduce stress levels, and contribute to a sense of calmness and rejuvenation (White et al. 2021). Attention restoration theory (ART) by Kaplan suggests that exposure to nature can reduce stress, improve mood, and restore work productivity (Berto 2014; Kaplan 1995; Van den Berg, Koole, and van der Wulp 2003). Exposure to environments that provide psychological distance from routine mental concerns combined with effortless, interest‐driven attention and supported by an environment of substantial scope can restore mental resources. Each of these three mechanisms that lead to mental restoration is found in natural settings (Hartig et al. 2003). Research on real‐life nature exposure supports that immersing in natural environments can be crucial in obtaining environmental advantages (Antonelli, Barbieri, and Donelli 2019). Research indicates that taking a stroll in nature led to a decrease in amygdala activity, whereas urban walking did not have the same effect. This suggests that spending time in nature can help reduce stress, and there is little evidence to suggest that being in urban environments increases amygdala activity (Sudimac, Sale, and Kuhn 2022). Another research revealed that being exposed to natural scenery, as opposed to urban settings, led to a reduction in the connection between certain parts of the distress network in the brain. Specifically, the link between the left insula and left subgenual anterior cingulate cortex was found to decrease. Furthermore, when compared to neutral stimuli, being in green spaces was connected to an increase in feelings of positivity and vitality (Imperatori et al. 2023). Furthermore, during short‐term exposure to nature, there were noticeable improvements in attention and cognitive flexibility, as indicated by rhythmic brain activities and increased functional connectivity among different brain regions. Specifically, the theta band showed significantly stronger power spectral density around the parietal region than other bands (Zhang, Wu, and Yang 2023). However, urban life and residency in some conditions, like rehabilitation centers for substance abusers, reduce patient's access to natural environments, depriving them of the benefits of being in natural environments.

Virtual reality (VR) is a revolutionary medium that can replicate extremely realistic virtual environments, providing an opportunity to deliver health benefits through simulated natural settings (Mattila et al. 2020). Various studies have revealed that using VR technology for nature immersion can have a positive impact on the psychological well‐being of individuals. A single session of VR is capable of reducing physiological stress, negative thoughts, and anxiety while promoting relaxation (Anderson et al. 2017). In particular, VR can be helpful in reducing stress and anxiety among individuals with mental health disorders (H. Kim et al. 2021; Malbos et al. 2020; Riches et al. 2023).

Therefore, VR has been considered a promising technology for virtual nature (Li et al. 2021). Due to reduced costs and increased portability, VR technology has emerged as a viable approach for healthcare and recovery (Wren et al. 2021). Relaxation is the primary function of virtual nature, including psychological and physiological relaxation (Riches et al. 2021). These relaxation effects can be detected through physiological indices (e.g., heart rate variability, electrodermal activity, and saliva cortisol) and self‐reported questionnaires (e.g., the Positive and Negative Affect Schedule and the State‐Trait Inventory) (Blum, Rockstroh, and Goritz 2019; Browning et al. 2020).

This study aims to examine the effects of VR on both affect and perceived stress, along with objective stress variables like heart rate, respiratory rate, and skin conductance in substance abusers undergoing detoxification. The participants in this study experienced 360° nature scenes, whereas the control group did not. By comparing the results of both groups, the study aims to provide insights into the potential benefits of using VR technology to alleviate stress and provide a more calming environment for individuals undergoing detoxification. Moreover, to determine whether the observed effects are due to nature or novelty, we included a group that experienced a 360° video of complex city scenes without natural elements as active control group.

## 2 Materials and Methods

**FIGURE 1:**
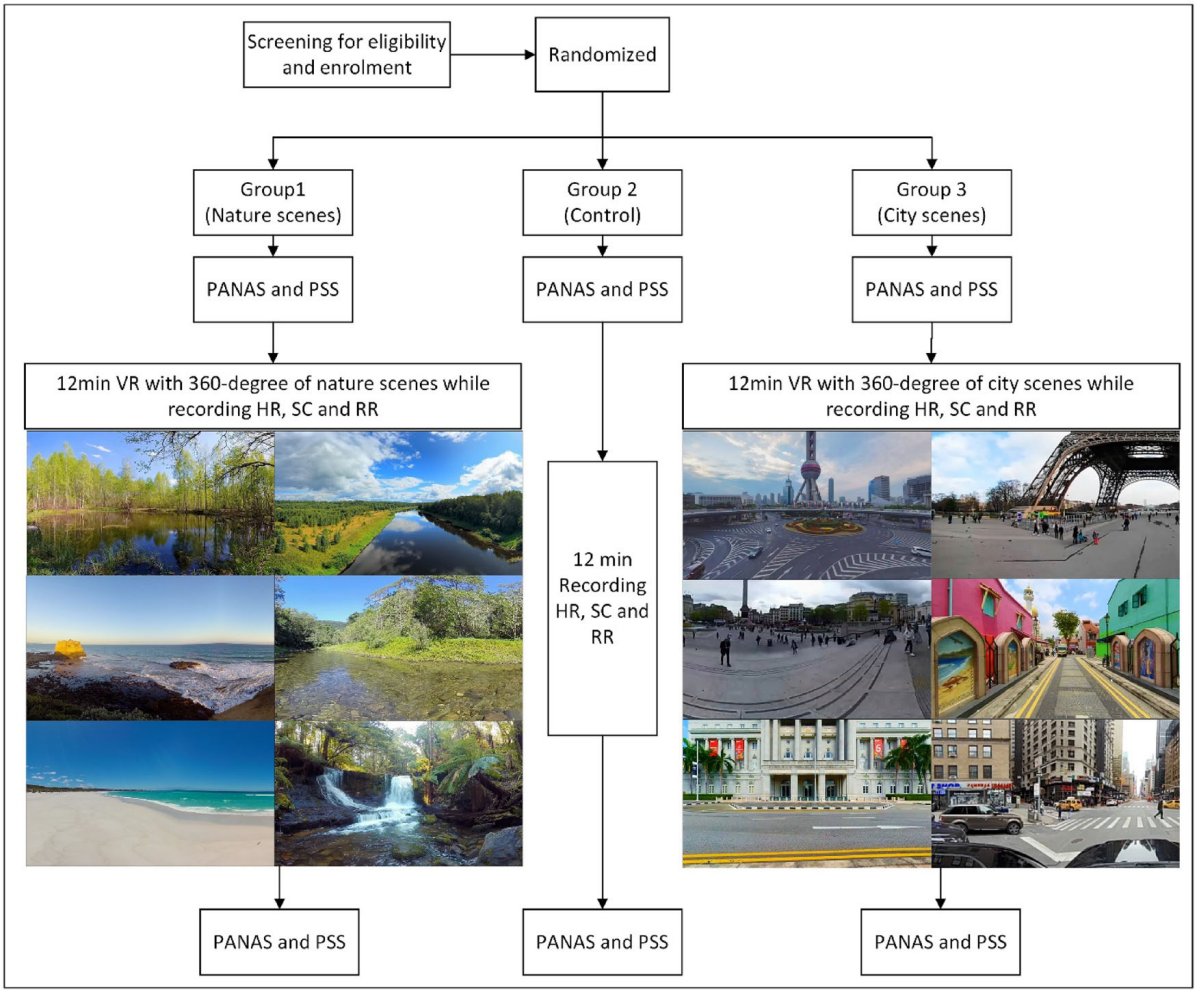
Flow chart of the study and example of 360° scenes. HR, heart rate; PANAS, positive and negative affect schedule; PSS, perceived stress scale; RR, respiratory rate; SC, skin conductance; VR, virtual reality.

This randomized, controlled, single‐blinded trial was conducted in 2023 with a 3‐arm design at a single center. Participants were recruited from a recovery center in Tehran, Iran. The flow chart of the trial is visualized in Figure 1.

### 2.1 Participants

The study focused on patients with substance dependency and undergoing detoxification at a rehabilitation facility for a duration of 10–14 days. Patients included in this study were diagnosed by a psychiatrist using standard psychiatric interviews on the basis of DSM‐5 criteria for opioid dependency. All participants were men between the ages of 20 and 60, undergoing detoxification, and without a history of psychotic or neurological disorders, epilepsy, or seizures. Patients were excluded if they did not complete questionnaires or sessions or experienced unusual symptoms like headaches. The sample size for this study was determined using GPower v3.1.1, with an effect size of 0.40, power of 0.8, and three groups. The calculated minimum sample size was 52 patients; ultimately, 60 patients (20 patients in each group) were included in the study.

### 2.2 Procedure

The randomization process involved assigning a unique number to the last 60 patients who were admitted to the center. Then, a number generator app randomly assigned the patients to one of three groups. This ensured that the group assignment was unbiased and based purely on chance. All 60 randomly selected individuals agreed to participate in the study. The researcher explained the study procedure to each patient, but they were not informed about the different scenarios in each group and the researcher's hypothesis that nature videos result in calmness. Prior to the study, written consent was obtained from all participants. A trained clinical psychologist conducted all procedures in a quiet room.

Initially, the participants completed self‐reported questionnaires, which included the perceived stress scale (PSS) and Positive and Negative Affect Schedule (PANAS). Afterward, the individuals in the experimental group were instructed to put on a Meta Quest 2 VR (Meta Technology, Liquid Crystal Display (LLC)) headset and wear devices that could measure their physiological responses. Then, a 360° video was played through the VR headset, whereas their physiological status was monitored and recorded every 1 min for 12 min. Free YouTube 360° videos were used to prepare VR images so the study could be repeated with similar videos. Examples of images in the videos used are available in Figure 1. This was done in a room completely free of external visual or auditory distractions. The headset featured a 5.5″ fast‐switching LCD at an 1832 × 1932 pixel per eye resolution. Group 1, also known as the nature group, watched 12‐min 360° high‐definition nature scenes. These nature scenes featured expansive views of water, jungle, and beach but did not include any animals or human elements. Sounds of nature and soothing music accompanied the scenes. Group 2, the control group, did not receive VR. They sat in the same room as the other groups and had their physiological data recorded, like the nature and city groups. Group 3, the city group, watched 12‐min 360° high‐definition scenes of cities. These scenes included large and expansive views of outdoor and indoor cityscapes with city sounds as well as human elements. Throughout the VR experience, respiratory rate, skin conductance, and heart rate were measured, and scales were filled out again after the VR experiences.

### 2.3 Tools

The study recorded demographic information of the participants, such as their age, education level, and marital status.

#### 2.3.1 Psychophysiological Measures

During the experiment, a Procomp‐5 Infiniti system (Thought Technology Ltd., Quebec, Canada) was used to obtain heart rate, skin conductance, and respiration data. An SC Flex/Pro sensor was used to measure skin conductance. It works by applying a tiny electrical voltage through two electrodes strapped to two fingers of one hand, creating an electric circuit where the subject becomes a variable resistor. As the subject becomes more stressed, the skin's conductance increases proportionally and vice versa (Choudhary, Trivedi, and Choudhary 2016). The heart rate was measured with a Blood Volume Pulse (BVP) sensor, where a higher heart rate indicates higher stress levels (Choudhary, Trivedi, and Choudhary 2016). In order to measure abnormal respiration rates, a Resp‐Flex/Pro sensor was employed. When a person is highly aroused, their respiration rate increases; conversely, when they are relaxed, it decreases (Omata and Tanabe 2015).

#### 2.3.2 Psychological Measures

PSS measures one's perception of life as unpredictable, unmanageable, and troublesome. The scale consists of 10 items, each evaluated on a Likert scale ranging from 0 (never) to 4 (often). A higher score on the scale indicates a greater level of perceived stress (Mazgelytė et al. 2021). The Persian version of this questionnaire has a reliability of 0.90 and a validity of 0.69 (Maroufizadeh, Zareiyan, and Sigari 2014). PANAS, on the other hand, is used to evaluate one's immediate affect. It consists of 20 questions, with half of the provided emotion words associated with positive affect (PA) (such as interested, alert, attentive, excited, enthusiastic, inspired, proud, determined, strong, and active) and the other half with negative affect (NA) (including distressed, upset, guilty, ashamed, hostile, irritable, nervous, jittery, scared, and afraid). Each question is answered on a 5‐point Likert scale ranging from “very slightly” to “very much.” The score for each emotion is calculated and combined to generate separate measures for PA and NA, which are independent of each other (Anderson et al. 2017). The Persian version of this questionnaire has a reliability of 0.87 and a validity of 0.77 (Ghorbanshiroudi and Abbas Ghorbani 2012; Sharifi, Bashardoust, and Emami 2012).

### 2.4 Statistical Analysis

The data are presented as mean with standard deviation (SD), and categorical data are reported as numbers with percentages. The normality of the data was assessed using the Shapiro–Wilk test. Demographic data were compared using one‐way ANOVA or the Kruskal–Wallis *H*‐test. The paired *t*‐test was used to compare approximately normally distributed continuous variables, whereas the repeated measure ANOVA was used to analyze continuous data. A *p* value of less than 0.05, two‐sided, was considered significant. The statistical analysis was conducted using R version 4.2.1 (R Foundation for Statistical Computing, Vienna, Austria).

## 3 Results

### 3.1 Participant's Background

All of the study participants were male, and their mean age was 36.95 (SD = 5.62) in the nature group, 38.05 (SD = 6.74) in the city group, and 40.10 (SD = 6.76) in the control group. The ANOVA results showed no significant difference in the mean age of the three groups (*F* = 1.24, *p* = 0.29). In the city and control groups, 15% of participants had a college education, whereas in the nature group, 20% had a college education. However, according to the Kruskal–Wallis test, the groups had no significant difference (*H* = 0.236, *p* = 0.88). In the nature and city groups, 30% of participants were married compared to 45% in the control group. Again, according to the Kruskal–Wallis test, there was no significant difference between the groups (*H* = 1.29, *p* = 0.52).

### 3.2 Psychological Scales

Scores of patients at baseline are compared with each other. As the results of ANOVA with the Bonferroni test showed, there was a significant difference between the nature and control groups in pretest of PA (PANAS) (*F* = 4.00; mean difference = −3.30; *p* = 0.02; *η* = 0.12). There was no significant difference between the other pretest scores between groups.

Table 1 displays the result of paired *t*‐test for comparing the mean scores of patients before and after intervention for each group. Paired *t*‐test assessing before and after VR experience indicated that PA (*p < *0.01), NA (*p < *0.01), and stress perception (*p < *0.01) significantly changed after exposure to a nature environment. There was no significant difference between city and control groups before and after a session.

**TABLE 1:** Results of paired *t*‐test for PSS and PANAS.

Groups		Pretest	Posttest	95% Confidence interval of the difference		*t*	*p*	Cohens *D*
		Mean (SD)	Mean (SD)	Lower	Higher			
Nature	PA	29.25 (2.76)	31.90 (3.35)	−4.39	−0.90	−3.17	< 0.01	−0.71
	NA	30.70 (3.14)	29.20 (3.39)	0.13	2.86	2.30	0.03	0.51
	PSS	28.65 (4.09)	26.20 (3.22)	0.67	4.22	2.89	< 0.01	0.64
City	PA	31.60 (3.74)	31.70 (3.40)	−0.46	0.26	−0.56	0.57	−0.12
	NA	32.05 (2.68)	31.80 (2.93)	−0.22	0.72	1.09	0.28	0.24
	PSS	30.15 (4.56)	29.85 (4.28)	−0.78	1.38	0.57	0.56	0.12
Control	PA	31.65 (3.75)	31.35 (4.13)	−1.81	2.41	0.29	0.76	0.06
	NA	32.20 (3.17)	31.10 (4.35)	−0.60	2.80	1.35	0.19	0.30
	PSS	29.50 (3.50)	30.25 (4.24)	−2.39	0.97	−0.88	0.38	−0.19

Abbreviations: NA, negative affect; PA, positive affect; PSS, perceived stress scale.

The ANOVA test with Bonferroni post hoc analysis was used to compare the mean scores of NA and PSS among different groups. There was no significant difference found among the groups for NA as determined by one‐way ANOVA (*F* (2,59) = 2.78, *p* = 0.07). However, for PSS, there was a significant difference between the groups (*F* (2,59) = 6.39, *p < *0.01). A Bonferroni post hoc test revealed that the mean score of the nature group was significantly lower in the posttest than the city (*p* = 0.15) and control groups (*p* = 0.006). An Analysis of covariance (ANCOVA) was conducted to assess differences in posttest results for PA, controlling for pretest scores. No significant differences were found in the posttest results between the groups [*F* (2,59) = 1.93, *p* = 0.15].

### 3.3 Physiological Data

**FIGURE 2:**
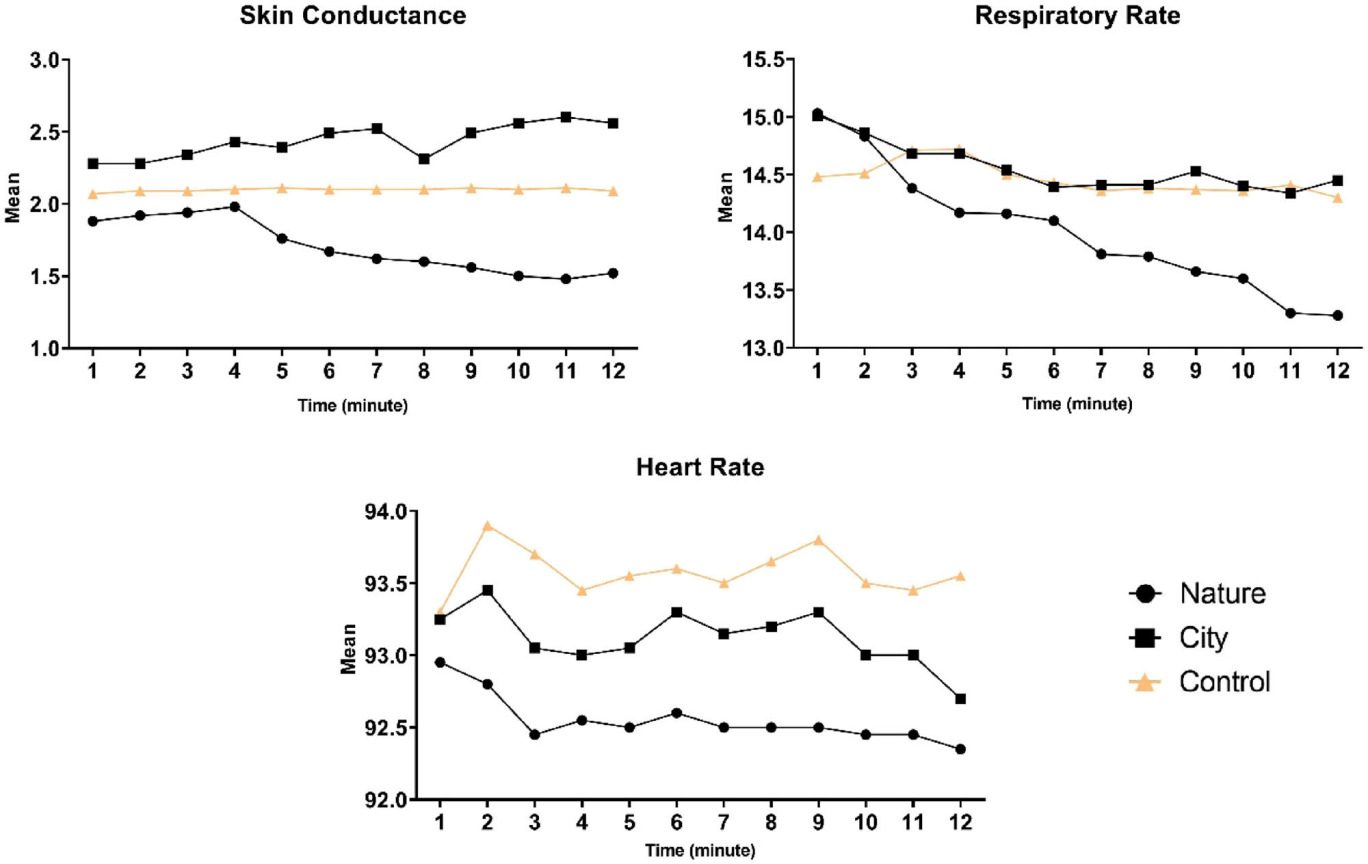
Each group's mean heart rate, respiratory rate, and skin conductance based on minutes of record are displayed using connecting lines and points.

The physiological mean scores for each minute of the experiment are visualized in Figure 2. A repeated measures ANOVA with Greenhouse–Geisser correction found a statistically significant difference in mean respiratory rate across 12‐time points in the nature group (*F* (2.65, 50.52)) = 8.17, *p < *0.01, np^2^ = 0.29) but not in city group (*F* (2.81, 53.36)) = 1.90, *p* = 0.14, np^2^ = 0.09) and control group (*F*(2.30, 43.78)) = 1.03, *p < *0.37, np^2^ = 0.05). Post hoc analysis with a Bonferroni adjustment revealed that respiratory rate showed a statistically significant decrease from Minutes 1 to 8 (1.23 (95% CI, 0.2–2.44), *p* = 0.04), Minute 9 (1.36 (95% CI, 0.11–2.61), *p* = 0.02), Minute 10 (1.42 (95% CI, 0.10–2.74), *p* = 0.02), Minute 11 (1.73 (95% CI, 0.32–3.14), *p* = 0.006), and Minute 12 (1.74 (95% CI, 0.19–3.31), *p* = 0.01). Moreover, there was a significant difference between Minutes 2 and 11 (1.53 (95% CI, 0.003–3.06), *p* = 0.04). Furthermore, Time × Group interaction revealed a significant difference among groups (*F* (5.93, 169.06) = 2.61, *p* = 0.02, np^2^ = 0.08).

Additionally, the results of skin conductance showed significant differences among the 12‐time points in the nature group (*F* (1.45, 27.67)) = 4.71, *p* = 0.02, np^2^ = 0.20) but not in city group (*F* (2.10, 40.03)) = 1.72, *p* = 0.18, np^2^ = 0.08) and control group (*F* (2.99, 56.93)) = 0.04, *p* = 0.98, np^2^ = 0.02). Post hoc analysis with a Bonferroni adjustment revealed that there was no significant difference between two‐time points. Furthermore, Time × Group interaction revealed a statistically significant difference between groups (*F* (5.20, 148.76) = 3.67, *p < *0.01, np^2^ = 0.11).

Analysis of heart rate shows no significant difference in nature group (*F* (1.79, 34.12)) = 0.24, *p* = 0.76, np^2^ = 0.01), city group (*F* (2.00, 38.18)) = 0.12, *p* = 0.89, np^2^ = 0.006), and control group (*F* (4.06, 77.16)) = 0.25, *p* = 0.91, np^2^ = 0.01). Moreover, Time × Group interaction revealed no significant difference between groups (*F* (4.90, 139.68) = 0.10, *p* = 0.99, np^2^ = 0.004).

## 4 Discussion

Participants were exposed to a virtual natural environment created by a VR device. The virtual environment consisted of both nature and city settings, each lasting 12 min. On the basis of the comparison between the pretest and posttest, the findings indicate that participants exposed to the natural setting experienced a significant decrease in scores on the perceived stress and NA. Additionally, they showed a significant increase in scores on PA. However, the posttest comparison among the groups showed that only perceived stress decreased significantly in the nature group in comparison to the city and control groups. Furthermore, physiological measures, such as respiratory rates and skin conductance, also decreased significantly in the nature group but with small effect size.

Notably, none of the subjects reported any unusual symptoms, such as dizziness, nausea, vomiting, headache, or visual disturbances. Although the study did not assess the implementation of VR, patients are likely to use it for relaxation and enjoyment on their own accord. Given the time constraints, VR could be a viable solution to alleviate stress as it reduces stress levels by providing an immersive video of natural settings, making the subjects feel like they are in a peaceful and calming environment (H. Kim et al. 2021).

The study found that VR experiences in natural environments can reduce psychological stress in detoxifying patients, as demonstrated by significant differences in self‐reported measures. This approach is consistent with the previous research (Calogiuri et al. 2022; Ho, Wu, and Yen 2023; Li et al. 2021; Spangenberger, Geiger, and Freytag 2022) and the findings of other researchers in ART (Berto 2005; Hartig et al. 2003). Looking at natural scenes was found to be more effective than looking at city scenes in reducing stress and improving affect during withdrawal. This study builds upon previous research on the impact of virtual nature on substance abuse. Like other studies using 360° videos of virtual nature, this study indicates that VR nature scenes can help substance abusers divert their attention from negative thoughts and withdrawal symptoms (Wilson and Scorsone 2021).

The findings show that similar to other studies using 360° videos of VR nature (Anderson et al. 2017), participants experienced an increase in PA and a decrease in NA when viewing VR nature scenes. Stress reduction theory suggests that natural landscapes promote well‐being because humans unconsciously identify with these landscapes for their evolutionary needs (Ulrich 1983). Simulated nature might primarily benefit from reducing NA, and according to the previous research (Van den Berg, Koole, and van der Wulp 2003) and ART (Kaplan 1995), nature can provide an escape from everyday demands and help to interrupt cognitive demands and maladaptive patterns of thought, potentially reducing NA without necessarily increasing PA (Golding, Gatersleben, and Cropley 2018; Van den Berg, Koole, and van der Wulp 2003).

Unlike other studies, our findings indicate that simulated nature exposure can have a positive impact on the overall affect rather than just reducing NA. This information could be important for future research and interventions aimed at improving mental health and well‐being. This aligns more with the findings from outdoor nature research than with simulated nature research (McMahan and Estes 2015; Neill, Gerard, and Arbuthnott 2019). Minimal variation in PA after a simulated natural encounter can be attributed to factors such as boredom or disconnection when viewing pictures or videos of environments (Brooks et al. 2017; Kjellgren and Buhrkall 2010). However, it is important to note that the present study kept the session time brief, which may have prevented participants from becoming bored.

We observed a gradual decrease in skin conductance and respiratory rates with exposure to nature scenes, whereas there was no significant change in these values for city and control groups. The respiratory system responds to mental stress by increasing the respiratory rate (Widjaja et al. 2013). The virtual nature experience has been found to lower the respiratory rate of substance abusers, which is a significant physiological stress index (Lin et al. 2011). Skin conductance is a measure of arousal or stress levels. The study showed that patients experienced increased relaxation after viewing natural scenes. Skin conductance is a symptom of the stress response due to sweat secretion by the eccrine sweat glands located all over the body (Westerink et al. 2020). It is widely used to measure the impact of built or natural environments on recovery after stress or cognitive tasks (Frumkin et al. 2017). Recent research has shown that a sudden increase in skin conductance can be a reliable indicator of the occurrence and timing of a stressful event. This increase in skin conductance level is associated with heightened arousal, influenced by cholinergic activity. Mental stress can also cause an increase in skin conductance level, further supporting the use of this measure as a reliable indicator of stress (Westerink et al. 2020). Such findings have important implications for understanding the physiological processes during stress and developing effective interventions to help individuals manage stress in their daily lives. The findings suggest that a decrease in a virtual nature environment compared with a city environment leads to decreased arousal, which may reflect feeling more relaxed and distract participants from their stressful thoughts. This finding shows that the virtual nature environment is beneficial for reducing the physiological stress of substance abusers.

According to our result, VR did not significantly change heart rate. A study conducted by Naylor et al. (2019) on healthy individuals demonstrated that heart rate significantly decreased after a VR intervention from pretest levels. An elevated heart rate is typically a sign of physiological arousal and is often accompanied by feelings of psychological stress and strain (Kao et al. 2014). Research conducted on the correlation between heart rate and stress levels has shown that a lower heart rate may indicate reduced stress or increased relaxation (Naylor et al. 2019). According to a study (Kennedy et al. 2015) heart rates were higher when substance abusers reported cravings. The high heart rate observed in substance abusers during our study may be attributed to physiological changes in the detoxification process, which may not decrease even after a VR experience, unlike skin conductance and respiratory rates. This finding suggests that substance abuse may have a significant impact on the body's physiological functioning, potentially leading to sustained heart rate elevation. However, it should be noted that the effects observed in other physiological variables of the study are not strong.

The potential advantages of using VR as a relaxation technique have been explored through various studies. One of the main benefits of VR relaxation is that it requires minimal effort in terms of attention and concentration, as the immersive nature of VR can help to alleviate stress and anxiety (Riches et al. 2023). Moreover, VR relaxation can be used independently, even in rehabilitation centers, making it a promising option for individuals seeking to manage their stress levels. Additionally, VR relaxation can be used as a standalone intervention, with fewer requirements from staff and less time‐intensive than other relaxation techniques. VR has many advantages for use in detoxification centers because it offers stress reduction and relaxation in psychological and physiological stress.

In this study, there are limitations that should be taken into consideration. First and foremost, patients cannot have multiple sessions during the day due to the treatment center's daily schedule. This means that the data collected may not be as comprehensive as it would have been if patients were able to have multiple sessions. It is recommended that future studies explore the impact of additional sessions. Second, the number of participants is limited due to recruitment challenges. This can potentially limit the generalizability of the findings. It is important to note that our study only included men. Future studies should investigate the effect of this intervention on women. Third, in this study, we did not utilize tools to measure immersion; therefore, it is recommended that future studies take this into account. Fourth, there is a lack of access to more precise tools to measure physiological status, which may impact the accuracy of the results. It is recommended that future research employ more precise methods to investigate the impact of this intervention and potential brain mechanisms. Finally, physiological measurements were taken during the intervention, not as pretest and posttest. Such an analysis could add additional results to the work. Despite these limitations, this study provides valuable insights into the application of VR in the treatment process of substance abusers.

## 5 Conclusion

The study found that a 12‐min VR experience that involves exposure to nature can significantly reduce both psychological and physiological stress levels. This is an important discovery, especially for individuals who may not have access to natural environments or for those who have substance abuse problems. For such individuals, a brief and isolated exposure to a 360° video of nature can be a beneficial alternative to visiting natural environments. However, the study emphasizes the need for further research to compare the effects of repeated exposure to virtual experiences with real‐life nature experiences in other populations. These findings have significant implications for developing interventions to reduce stress and improve mental health.

## Author Contributions

**Shahab Lotfinia**: conceptualization, methodology, writing–original draft, writing–review and editing, formal analysis, project administration. **Aram Yaseri**: conceptualization, resources, writing–original draft, visualization. **Pardis Jamshidmofid**: resources, investigation, supervision. **Fatemeh Nazari**: supervision, resources, writing–review and editing. **Homa Shahkaram**: methodology, investigation, resources. **Jafar Sarani Yaztappeh**: software, validation. **Amir Sam Kianimoghadam**: supervision, methodology, conceptualization, writing–review and editing, funding acquisition, data curation.

## Ethics Statement

The study protocol was approved by the Ethics Committee of the Shahid Beheshti University of Medical Science (IR.SBMU.RETECH.REC.1402.303) and Iranian Registry of Clinical Trials (IRCT20220316054306N2).

## Consent

This manuscript has been approved for publication by all authors.

## Conflicts of Interest

The authors declare no conflicts of interest.

### Peer Review

The peer review history for this article is available at https://publons.com/publon/10.1002/brb3.70084.

## Data Availability

The datasets generated and analyzed during the current study are not publicly available to protect patient confidentiality but are available from the corresponding author upon reasonable request.
